# Development of a Multimodal, Personalized Intervention of Virtual Reality Integrated Within Physiotherapy for Patients With Complex Chronic Low-Back Pain

**DOI:** 10.1089/jmxr.2023.0002

**Published:** 2024-01-25

**Authors:** Syl Slatman, Tjitske Groenveld, Raymond Ostelo, Harry van Goor, J. Bart Staal, Jesper Knoop

**Affiliations:** 1 Department of Surgery, Radboud University Medical Center, Nijmegen, The Netherlands.; 2 Department of Musculoskeletal Rehabilitation Research Group, School for Allied Health, HAN University of Applied Sciences, Nijmegen, The Netherlands.; 3 Department of Biomedical Signals and Systems Group, Faculty of Electrical Engineering, Mathematics and Computer Science, University of Twente, Enschede, The Netherlands.; 4 Department of Health Sciences, Faculty of Science and Amsterdam Movement Science Research Institute, Vrije Universiteit, Amsterdam, The Netherlands.; 5 Department of Epidemiology and Data Science, Amsterdam UMC location Vrije Universiteit and Amsterdam Movement Sciences, Musculoskeletal Health, Amsterdam, The Netherlands.; 6 Department of IQ Healthcare, Radboud Institute for Health Sciences, Radboud University Medical Center, Nijmegen, The Netherlands.

**Keywords:** virtual reality, physiotherapy, chronic low-back pain, cocreation, intervention development

## Abstract

**Background:**

Chronic low-back pain (CLBP) is the leading cause of years lived with disability. Physiotherapy is the most common treatment option for CLBP, but effects are often unsatisfactory. Virtual reality (VR) offers possibilities to enhance the effectiveness of physiotherapy treatment. Primary aim was to develop and test a personalized VR intervention integrated within a physiotherapy treatment for patients with CLBP.

**Methods:**

This study describes an intervention development process using mixed methods design that followed the Medical Research Council (MRC) framework. This involved a cocreation process with patients, physiotherapists, and researchers. A draft intervention was constructed based on a literature review and focus groups, and subsequently tested in a feasibility study and evaluated in focus groups. Focus group data were analyzed using thematic analysis. This intervention development process resulted in a final intervention.

**Results:**

Focus group data showed that VR and physiotherapy can strengthen each other when they are well integrated, and that VR needs to be administered under the right conditions including flawless technology, physiotherapists with sufficient affinity and training, and the right expectations from patients. The draft intervention was considered feasible after evaluation by four patients and three physiotherapists and was further complemented by expanding the training for physiotherapists and improving the protocols for physiotherapists and patients. The final intervention consisted of a 12-week physiotherapy treatment with three integrated VR modules: pain education, physical exercise, and relaxation.

**Conclusion:**

Using the MRC framework in cocreation with the end users, a personalized VR intervention integrated within a physiotherapy treatment for patients with CLBP was developed. This intervention was found to be feasible and will subsequently be evaluated for (cost-)effectiveness in a cluster randomized controlled trial.

## Introduction

Chronic low-back pain (CLBP), defined as back pain that persists for 3 months or longer,^1^ is the most frequent global chronic pain condition^2^ and is a leading contributor to disability and disease burden.^3^ CLBP is also associated with psychological complaints, social restrictions, and high societal costs.^1^ Like comparable chronic musculoskeletal pain (CMP) conditions, CLBP is a complex problem because of an interplay of biological, psychological, and social factors.^4^

Current treatment options for patients with CLBP vary and include both pharmacological and nonpharmacological possibilities.^5^ Physiotherapy is the most common nonpharmacological treatment, usually consisting of a combination of (home) physical exercises and pain education.^6,7^ However, effects of physiotherapy seem to decrease or even disappear after stopping treatment.^8^ Owing to the frequent presence of psychosocial factors in patients with CLBP, an integrated biopsychosocial approach is recommended.^9,10^

However, physiotherapists often struggle with providing psychosocial treatment elements to patients with CLBP,^11^ due to, for example, physiotherapists' skills and patient expectations.^12^ Another limitation in CLBP physiotherapy treatment is that patients seem to be unable to keep performing their exercises at home.^13^

Virtual reality (VR) could support patients with CLBP in treatment adherence and guide physiotherapists in providing biopsychosocial treatment. This way VR potentially optimizes the effects of physiotherapy on both the short and long term. Specifically in CLBP, therapeutic VR provides multiple possibilities to support physiotherapists in optimizing their tailored treatment. In VR, a user interacts with a computer-simulated, immersive, three-dimensional world.^14^ Therapeutic VR could be used for a variety of treatment goals, including relaxation,^15^ graded exposure,^16^ and distraction.^17^

For acute pain, VR seems to be an effective treatment modality,^18^ whereas for chronic pain this is less apparent.^19^ Prior studies mainly focused on stand-alone therapeutic VR, and showed that this could be an effective treatment option for patients with CLBP.^20,21^ However, it has been suggested that an integration of VR within the CLBP physiotherapy treatment could further enhance its effectiveness on clinical and adherence outcomes, in favor of VR as stand-alone treatment next to physiotherapy.^20,22^ This integration comprises, for example, prescription of the VR exercises by the physiotherapist based on patients' needs and preferences (i.e., personalization). Nevertheless, it should be noted that this integration of VR in the physiotherapy treatment was not empirically examined in clinical practice yet.

The aim of this study was, therefore, to develop a personalized VR intervention integrated within a physiotherapy treatment for patients with CLBP through a cocreation process. In the development of complex interventions, a cocreation process is advised to develop an intervention that fits differences in needs and interests of stakeholders.^21^ Cocreation entails development in collaboration with the end users rather than development solely for the end users, actively involving stakeholders in the process of intervention development.^22^ This intervention will subsequently be evaluated for (cost-)effectiveness in a cluster randomized controlled trial (RCT).^23^

## Materials and Methods

### Design

This mixed-methods study is part of the VARIETY project, which aims to investigate the (cost-)effectiveness of a personalized VR intervention integrated within a physiotherapy treatment for patients with CLBP in a large cluster RCT.^23^ This study, which was conducted following the Medical Research Council (MRC) framework^24^ and the best practice framework developed by the Virtual Reality Clinical Outcomes Research Experts (VR-CORE) international working group,^25^ aimed at developing of our intervention in cocreation with the end users.

**FIG. 1.**
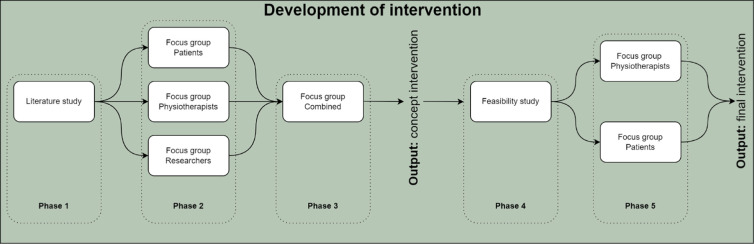
Development process of intervention.

The MRC framework consists of core elements and four not necessarily consecutive stages, including the development, feasibility testing, evaluation, and implementation of an intervention. This study covered the stages development and feasibility, which are represented by phases 1–3 (development) and phases 4 and 5 (feasibility) in Figure 1. The stages evaluation and implementation will be described in a following study.^23^

The best practices framework of the VR-CORE group states three main phases with accompanying methodologies in the research of VR. This study is a combination of a VR1 and VR2 study, focusing on content development and early feasibility.

Ethical approval was obtained by the research ethics committee of the HAN University of Applied Sciences (HAN ECO: 319.01/22) and the research was conducted according to the principles of the Helsinki Declaration and in accordance with Dutch guidelines, regulations, and acts (Medical Research involving Human Subjects Act, WMO).

### Procedures

#### Literature review (phase 1)

The aim of this literature review was to examine current “state-of-the-art” CLBP care in primary care physiotherapy and identify recommendations regarding VR. A pragmatic literature review was conducted. We preferred this type over a systematic approach, because of its convenience and usefulness for the purpose of this study.^26^ The review was carried out by utilizing and crosslinking the Dutch physiotherapy guideline for patients with LBP,^27^ supplemented by a new literature search using PubMed to identify international research and clinical guidelines on VR interventions for patients with CLBP.

Peer-reviewed studies from January 2010 up to March 2022 in English or Dutch were included. The search included combinations of the following keywords: “chronic low back pain” or “CLBP,” “virtual reality,” and “physiotherapy” or “physical therapy.” Based on information gathered in the literature search, supplemented with clinical knowledge and experience of the research team, a framework for the draft intervention was developed.

#### Focus groups (phases 2, 3, and 5)

Focus groups were used for data collection in phases 2, 3, and 5, to collect opinions and suggestions with regard to the draft intervention. For reporting, the consolidated criteria for reporting qualitative research checklist were used.^28^ The focus group sessions followed the divergence–convergence model,^29^ in which the focus groups of phases 2 and 3 had an inductive structure and the focus group of phase 5 a deductive structure.

Semistructured interview guides with open-ended questions were constructed, as shown in the moderator guide (Appendix Table A1).^30^ All focus group sessions were conducted and audio recorded online using Microsoft Teams (Microsoft Corporation, Redmond, WA, USA) and were moderated by one of the researchers (S.S.). The moderator followed a semistructured interview guide, ensured that all patients were actively involved in the discussion, and generated interaction between participants. The four sessions had a median duration of 66 (range: 45–82) min.

The final focus groups session was conducted similar to those in phases 2 and 3. A new semistructured interview guide was constructed based on the focus groups in phases 2 and 3, the feasibility study in phase 4, and the literature.^31^

#### Feasibility study (phase 4)

Before the start of the feasibility study, physiotherapists received a single 2-h group training on the draft intervention, including the personalization of the intervention, integration of VR within physiotherapy treatment, and practical use of VR headsets. After this training, the physiotherapists could get familiar with the VR headset and go through the different VR modules during 1 week without patients.

Patients referred to a participating physiotherapist practice because of CLBP were screened for study participation by the physiotherapist during the patient's intake. The physiotherapist provided eligible patients with verbal and written information and obtained informed consent. Subsequently, patients were contacted by the coordinating researcher and instructed to complete questionnaires at baseline and directly after the intervention at 12 weeks.

Enrolled patients with CLBP received the draft intervention of VR integrated in physiotherapy.

Demographic information about the patients was gathered at baseline. The following Dutch questionnaires were administered at baseline (T0) and directly after the intervention (T1): physical functioning using the Oswestry Disability Index (ODI),^32^ pain intensity using the numeric rating scale (NPRS),^33^ pain-related fears using the fear avoidance beliefs questionnaire, physical activity subscale (FABQ-PA)^34^ and pain catastrophizing scale (PCS),^35^ general effect using global perceived effect,^36^ problems with activities using the patient-specific complaints questionnaire,^37^ and pain self-efficacy using the pain self-efficacy questionnaire.^38^

In addition, physiotherapists were instructed to report possible adverse events (AEs), treatment modalities, and duration of the physiotherapy treatment after each physiotherapy session. Last, used VR modalities and duration of VR usage per day were extracted from an online dashboard in which used VR treatment modalities and duration were registered.

### Participants

#### Focus groups (phases 2, 3, and 5)

Purposive sampling was used to compose three homogeneous focus groups for phase 2. Patients were recruited through the newsletter of a low-back pain patient organization (de Wervelkolom; https://www.ruginfo.nl/), a previous study with a similar patient population, and physiotherapists who use therapeutic VR. Inclusion criteria for patients were experience with therapeutic VR, and having CLBP. Physiotherapists were recruited through direct contact and social media posts. Inclusion criteria were experience with therapeutic VR and practicing as a physiotherapist. Researchers were recruited through direct contact using one inclusion criterion: participated before in VR studies. A heterogeneous focus group for phase 3 was created through inviting patients, physiotherapists, and researchers participating in phase 2.

All participating patients and physiotherapists in phase 4 were asked to participate in the focus groups of phase 5.

#### Feasibility study (phase 4)

Physiotherapists for the feasibility study were recruited using convenience sampling through directly contacting physiotherapy practices in the network of the researchers. The only inclusion criterion was practicing as a physiotherapist. Physiotherapists recruited patients for the feasibility study using the following inclusion criteria: age 18–80 years, CLBP >3 months as reason to visit the physiotherapist, absence of “red flags” or signs of specific LBP,^27^ combination of severe disability (ODI score ≥40^32^), and severe pain (NPRS ≥5^33^).

Participants were excluded if they had severe comorbidity that would substantially hinder the physiotherapy treatment, had a planned diagnostic or invasive treatment procedure (e.g., injection, nerve block, or operation) for their CLBP in the next 3 months, lacked comprehension of Dutch language, were not able to use VR (e.g., because of epilepsy, open wounds on face, or severe visual impairment), and had no e-mail address and wi-fi connection at home.

Initially, the inclusion criterion of ODI score was set for 40 or higher, as this score singly is associated with severe disability.^32^ However, during the feasibility study, physiotherapists stated that many patients under-reported their disability and, therefore, they had to exclude many patients who they would consider eligible. Therefore, we decided to lower the ODI score to 30 or higher. Previous studies among patients with CLBP found that average ODI scores were ∼23^39,40^ and the cutoff ODI score of 30 or higher was used in previous studies. It should be noted that an ODI score of 30 or higher combined with NPRS ≥5 is still expected to identify patients with complex CLBP in primary care physiotherapy.

### Data analysis

#### Literature review (phase 1)

Studies found during the pragmatic literature review were screened by S.S. for usefulness regarding the development of a VR intervention integrated within physiotherapy. Studies (e.g., guidelines, reviews, or RCTs) were deemed eligible for inclusion if they included information on VR treatment for CLBP, physiotherapy treatment for CLBP, or the integration of VR in physiotherapy care. Relevant features regarding intervention content (VR and physiotherapy), intervention duration, integration of VR therapy within physiotherapy treatment, location of administration, and target population were extracted and considered for the development process.

#### Focus groups (phases 2, 3, and 5)

The content of the focus groups was transcribed using Amberscript (Amberscript, Amsterdam, The Netherlands). The transcripts were analyzed by two independent researchers (S.S. and T.G.) in Atlas.ti version 22 (Atlas.ti, Berlin, Germany) using inductive thematic analysis.^41^ The research question used was “how can the intervention be improved?” For phase 5, the research question was somewhat specified: “what were strengths of the draft intervention used in the feasibility study and how can the intervention be improved?”

Both researchers independently coded the transcripts for meaningful improvements of the intervention. After each transcript, the codes were discussed by the two researchers until consensus was reached, which was repeated for all transcripts. Subsequently, the identified codes were categorized into themes. The final codes and themes were discussed with all other researchers until consensus was reached.

#### Feasibility study (phase 4)

The feasibility of the intervention was reported descriptively using the physiotherapists' treatment registration, results on outcome variables on T0 and T1, and the VR data about used treatment modalities and duration. Moreover, possible improvements for the intervention and trial were registered during the feasibility study by the coordinating researcher (S.S.).

## Results

### Phase 1: Literature review

The results of the pragmatic literature search yielded one relevant clinical guideline,^30^ six RCTs,^15,21,22,42–44^ three (systematic) reviews,^45–47^ two other studies,^23,48^ and one book^49^ on VR and/or CLBP.

In summary, Swart et al. describe in the Dutch physiotherapy guideline for patients with LBP that a treatment duration of ∼12 weeks is most fitting for complex patients,^27^ and this treatment duration showed the best results in VR interventions for CLBP.^46^ Several types of VR programs in the literature seem beneficial for patients with CLBP, including pain education,^43,52^ activation,^48^ relaxation,^15^ and distraction.^44^ Combination of these modules might offer a valuable contribution to usual physiotherapy.^43,46^

There are various opinions on using either specifically designed VR applications or commercially available applications.^48^ Specifically designed applications offer the possibility to target specific movements, pain sites, and with that a specific population. Whereas commercially available apps are more likely to be applicable to people with a range of functional impairments and are more accessible. Several studies state that the integration of VR modules within physiotherapy could be more effective than stand-alone VR modules.^20,53^ This is in line with the physiotherapy guideline that states that therapeutic eHealth could support physiotherapy treatment.^27^

Regarding the location of VR therapy use, both use at the physiotherapy practice as use at home has been described.^48,52^ Benefits of VR therapy at the physiotherapy practice are professional assistance of a physiotherapist and guidance in translating exercises to daily life. Possible benefits of at-home VR therapy are accessibility and taking exercises to the patients' own environment. Two studies describe the importance of exposure and opportunities of VR therapy to expose patients in a controlled and playful environment.^45,49^ Within patients with CLBP, VR interventions seemed to be more effective in more complex patients.^42,47^

#### Draft intervention

Based on the results of the pragmatic literature review, a framework for the intervention was constructed. The draft intervention comprised a personalized, 12 weeks, intervention of VR therapy integrated in physiotherapy. The VR therapy contained three modules (pain education, physical exercise, and relaxation). The physiotherapy treatment contained of guideline-based usual care. An integrated, shared-decision-making approach with home-based VR treatment combined with physiotherapy sessions was deemed most appropriate.

### Phases 2 and 3: Focus groups

#### Demographics

Demographic information of the participants in the focus groups is described in Table 1. Physiotherapists who participated in the focus group held a master's degree for psychosomatic (*n* = 3), manual (*n* = 2), or occupational (*n* = 1) physiotherapy. Researchers who participated in the focus group held functions including (associated) professor (*n* = 3), senior researcher (*n* = 3), or physiotherapy researcher (*n* = 2). The combined focus group (*n* = 5) of phase 3 comprised patients (*n* = 2), physiotherapists (*n* = 2), and researchers (*n* = 1) who participated in the focus groups of phase 2.

**Table 1.** Demographics of Focus Group Participants of Phase 2

	Focus group patients (***n*** = 4)	Focus group physiotherapists (***n*** = 5)	Focus group researchers (***n*** = 8)
Gender (female), *n* (%)	3 (75)	4 (80)	4 (50)
Age (years), median (range)	44 (29–73)	33 (29–46)	48 (27–64)
Duration of complaints (years), median (range)	14 (12–30)	—	—
Experience with VR (months), median (range)	2 (1.5–6)	28 (6–90)	—
Experience as physiotherapists (years), median (range)	—	11 (6–23)	—
Experience as researcher (years), median (range)	—	—	15 (1–30)

VR, virtual reality.

#### Thematic analysis

**FIG. 2.**
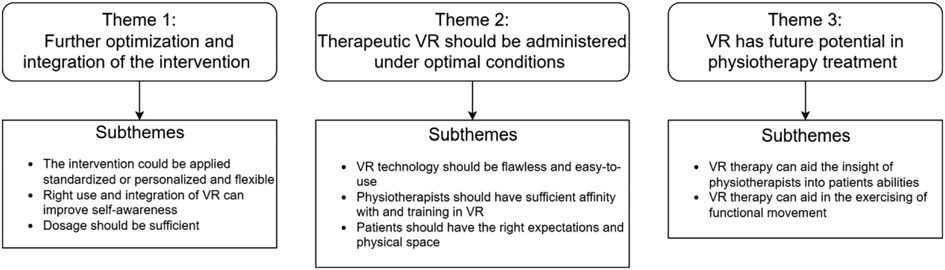
Identified themes and subthemes. VR, virtual reality.

A total of three overarching themes with multiple subthemes are shown in Figure 2.

##### Theme 1: further optimization and integration of the intervention

###### Subtheme: the intervention could be applied standardized or personalized and flexible

An important topic regarding the optimization of the intervention design was the question to what extent the intervention should be standardized or leave room for personalization. Some participants advocated for a strict protocol for the physiotherapists to apply the intervention, to make sure physiotherapists exactly know how to administer the intervention:

I would use a decision tree-like protocol, that allows you to choose one module using certain indications which are quite clear. (physiotherapist 3)

Others had a different view and proposed a minimal protocol with room for physiotherapists to personalize the intervention:

I don't even think you should use a standard set [of VR modules] that everyone should get, but you have a number of modules available and based on analysis of your physical examination, history taking, and other tests you make a choice. (researcher 5)

One possible suggested way to personalize the intervention was through shared decision making:

The best of course would be, if you want to imitate clinical physiotherapy practice as much as possible, to leave it to the therapist in consultation with the patient. (physiotherapist 5)

The method of applying VR modules was discussed and a more parallel approach in which it is possible to alternate between VR modules was preferred:

I would not like to first relax for a week, then exercise for a week and then receive pain education. I would like to use them mixed together. (patient 4)

###### Subtheme: right use and integration of VR can improve self-awareness

Several methods to improve self-awareness of patients were discussed. First, it was advised to have patients repeat learned skills and knowledge during the intervention:

When thinking about pain education…you can't do that once. I think you need to repeat and repeat and repeat that. (researcher 8)

Moreover, self-awareness could be improved by incorporating learned skills and knowledge from VR into daily life:

And also look at how you can use that in daily life, in an exercise or in a psychological exercise or in a physical exercise. (physiotherapist 1)

Finally, it was mentioned that self-awareness could be improved through feedback of the physiotherapist on experiences attained in VR.

…that if people have an experience, you discuss it in a certain way, so a learning effect arises. (researcher 5)

###### Subtheme: dosage should be sufficient

A total intervention duration from at least 6 to 12 weeks for the total intervention was considered sufficient by participants. The maximum duration of a single VR session was discussed to be ∼30 min:

We have also had people who practiced for hours, but they say that cybersickness increases after 30 minutes. So we do use 30 minutes as a maximum, also internationally. (researcher 1)

I also really think you should use it [VR] longer, so that's my main takeaway. That you can't really have a result in six weeks. (patient 1)

##### Theme 2: therapeutic VR should be administered under optimal conditions

Different conditions were considered important in the context of the VR applications, the role of the physiotherapist, and the role of the patient.

###### Subtheme: VR technology should be flawless and easy to use

Participants stated that functioning technology was crucial in using therapeutic VR. Common technological obstacles that were mentioned included slow technology, limited battery life, and lack of explanation in VR applications. Participants mentioned that decent technological support was warranted.

Some potential beneficial elements of VR exercises were mentioned that might optimize the intervention. Patients said that a reward system in VR exercises might stimulate treatment adherence. Also, the possibility of using VR in different postures (e.g., sitting or lying down) and having variation within and between VR applications were mentioned as facilitators for VR use:

So the variation yes, change something about the environment or make it more challenging, so you feel like you level up or something. (patient 4)

Multiple participants stated that it is important to try to prevent side effects due to VR use.

Your eyes receive the signal that you are on a road and you have to dodge cars, but the bike you are on is stationary. So, what you see and what your body is doing, if those are not corresponding, you could get sick. (patient 4)

###### Subtheme: physiotherapists should have sufficient affinity with and training in VR

Participants mentioned that it was necessary for physiotherapists administering therapeutic VR to be skilled and trained in doing so (e.g., setting realistic expectations).

The attitude, the knowledge and the skills in that area [VR] are important, because otherwise they [physiotherapists] might administer the VR intervention, but not in the best way possible. (researcher 5)

Receiving sufficient preparatory information was considered important, including instructions about VR use and its side effects, but also about administering and communicating about the intervention in general. Having peer support groups for physiotherapists was thought to be helpful. To make sure the intervention is administered properly, physiotherapists should have sufficient time to do so.

Besides certain skills, physiotherapists should be motivated and enthusiastic to use VR in their physiotherapy treatment.

We have had physiotherapists who could not handle VR themselves because they would get too dizzy. Patients complained a lot about this, since their physiotherapists did not understand what they were doing. (researcher 2)

###### Subtheme: patients should have the right expectations and physical space

Managing expectations of patients was thought to be very important in optimizing conditions for patients to receive the intervention.

What you have to take into account, is the fact that VR is a very hyped treatment. And a hyped treatment could induce a serious placebo effect, but also a nocebo effect. (researcher 1)

Communication of physiotherapist to patient was deemed important. Patients should receive adequate information from their physiotherapist about the VR headset, VR modules, and intervention in general.

Patients could benefit more from the intervention in the right setting. This could be achieved by having a space to themselves or to use the VR modules in a quiet surrounding, possibly using headphones.

##### Theme 3: VR has future potential in physiotherapy treatment

###### Subtheme: VR therapy can aid the insight of physiotherapists into patients abilities

To gain insight into the patients' performances, several new applications of VR technology were suggested, including the possibility to use biofeedback, measure range of motion, and examine a patient's load and capacity using VR.

Motion sensors yes, to map movement and using biofeedback to visualize the load and loadability of a patient. (physiotherapist 4)

###### Subtheme: VR therapy can aid in the exercising of functional movement

Several possibilities were advised to incorporate VR in the exposure module of the intervention, including the use of pain education and activating VR modules.

When you have the exposure module and then integrate VR into it, for example by using Reducept in step two [education] and for example by using SyncVR Fit in step four [generalization], then you can integrate it. (physiotherapist 1)

Also body region-specific exercises in VR were thought to be of added value, like exercises for core stability or back muscles.

…a little more for the spine, flexion and extension…I don't know if that exists in VR, perhaps like Pilates, a little bit of that learning to move, a little more focused on the spine specifically. (physiotherapist 3)

Finally, it was suggested to incorporate a VR module to improve the quality of movement of patients.

It would be nice if people start moving more nicely. (physiotherapist 3)

### Phase 4: Feasibility study

#### Demographics

The feasibility study was conducted by four physiotherapists (three women, one man) with the following physiotherapy specializations and master's degrees: manual therapy (*n* = 2), psychosomatic physiotherapy (*n* = 1), and none (*n* = 1). One of the physiotherapists had previous experience with therapeutic VR (2 years). During the feasibility study, one of the physiotherapists started a new job and stopped participating.

**FIG. 3.**
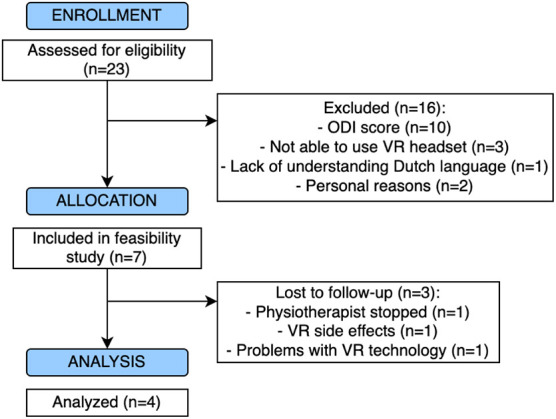
Flow of patients in feasibility study. ODI, Oswestry Disability Index.

A total of 23 patients were deemed eligible for participation in the feasibility study (Fig. 3). In total, seven patients were enrolled of which four completed the 12-week intervention. These four patients (two women) had a median age of 56 years and a duration of CLBP of 14 years (Table 2). The patients who completed the intervention showed improvements on all outcome measures except for physical activity (Table 3).

**Table 2.** Demographics of Feasibility Study Patients (*n* = 4)

Gender (female), *n* (%)	2 (50)
Age (years), median (range)	56 (20–70)
Duration of complaints (years), median (range)	14 (4–30)
Comorbidities, yes (%)	3 (75)
Occupation, *n* (%)	
Yes	1 (25)
No	1 (25)
Retired	2 (50)
Education level, *n* (%)	
Higher	1 (25)
Intermediate	2 (50)
Lower	1 (25)
Undergone diagnostic procedures past 6 months, yes (%)	3 (75)
Undergone therapeutic procedures past 6 months, yes (%)	4 (100)
Medication use, yes (%)	4 (100)
Experience with therapeutic VR, yes (%)	0 (0)
Experience with VR for entertainment, yes (%)	1 (25)

**Table 3.** Pre- and Post-Treatment Outcome Measures of Feasibility Study

	T0	T1
ODI	43 (32–50)	20 (0–56)
NPRS	8 (7–8.5)	7 (4–8)
FABQ-PA	14 (10–14)	13 (8–14)
PCS	28 (12–44)	21 (3–31)
Physical activity (minutes/week)	405 (70–900)	240 (210–840)
PSEQ	28 (19–34)	34 (30–47)
EQ-5D		
Quality of life (index value)	0.8 (0.7–0.8)	0.8 (0.7–0.9)
Health status	4 (3–5)	7 (4–7)
GPE		
Recovery	n/a	6 (5–6)
Satisfaction	n/a	6 (5–7)
CEQ		
Credibility	29 (0–32)	n/a
Expectancy	31 (0–33)	n/a

All values are reported as median (range) unless specified otherwise.

CEQ, Credibility/expectancy questionnaire; EQ-5D, EuroQol 5 dimensions; FABQ-PA, fear avoidance beliefs questionnaire, physical activity subscale; GPE, global perceived effect; n/a, not applicable; NPRS, pain intensity using the numeric rating scale; ODI, Oswestry Disability Index; PCS, pain catastrophizing scale; PSEQ, pain self-efficacy questionnaire.

Patients received on average 6.3 usual physiotherapy treatments. Treatment modalities differed between physiotherapists and included manual therapy (48%), exercise therapy (48%), pain education (28%), and psychosomatic therapy (24%). Median VR use was 58.3 min per week (range: 30.3–126.8). Among the three VR modules in the intervention, SyncVR Relax & Distract was used most (67%), followed by Reducept (17%), and SyncVR Fit (16%). No AEs were reported.

### Phase 5: Focus groups

#### Demographics

The focus group of patients (*n* = 2) included two men, aged 67 and 70 years, who were suffering from CLBP for 10 and 30 years, respectively, and completed the draft intervention. The focus group of physiotherapists comprised the three physiotherapists who administered the draft intervention during the feasibility study.

#### Focus group results phase 5

Thematic analysis of the two focus groups from phase 5 identified similar themes and subthemes as found in the focus groups of phases 2 and 3, confirming the importance of these recommendations. Some themes were supplemented with new insights. A specific recommendation from patients was to have a better explanation about the VR modules from their physiotherapists.

Well, I might have misunderstood those, but I couldn't get them to function to be honest. I also tried to do the Sudoku game, but I don't know, I thought the manual and explanation were very brief and limited. (patient 1)

Participants confirmed and further stressed the importance of the possibility to personalize VR modules to the needs and wishes of the patient and their therapist.

I would like to have [VR] exercises that would fit with my abilities and inabilities to function…and I think maybe you can focus a bit more in the future, to see whether someone has physical problems or someone has mental problems, let's say memory problems, and say: this program will benefit you the most. (patient 1)

Finally, it was suggested to further expand the VR modules toward, for example, motor imagery training.

But I think, if you look at the applicability [of VR] to pain, there are many more possibilities in terms of content…visualization is different in people with chronic pain. And yes, I think those things could be even better in the future. And motor imagery training, you know what I mean right? (physiotherapist 3)

### Final intervention

After evaluation of phases 4 and 5, several improvements for the final intervention were suggested. The training for physiotherapists was extended and split in two parts of ∼90 min (i.e., VR software and hardware, and intervention and research). The protocol for physiotherapists and explanation for patients was revised based on input from the participants and physiotherapists in the feasibility study (e.g., more extensive manual on VR use).

#### Intervention content

**FIG. 4.**
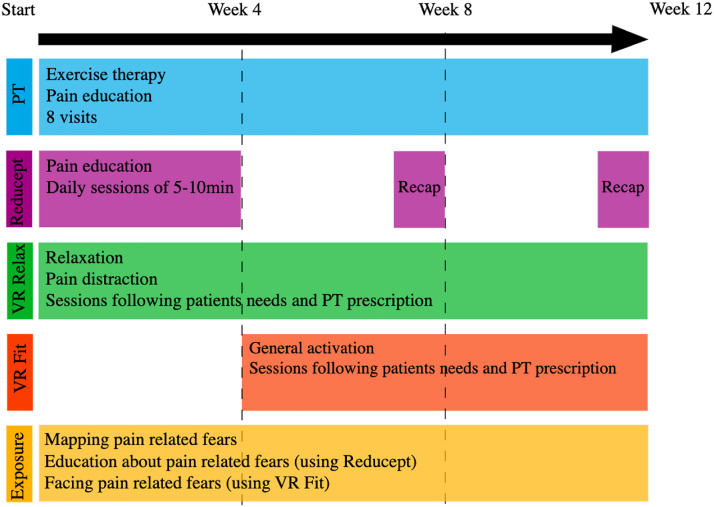
Schematic representation of final intervention. PT, Physiotherapist.

Based on the findings from the feasibility study and last focus groups, final adjustments were made to the draft intervention resulting in the final intervention (Fig. 4). The final intervention consisted of a personalized intervention of VR therapy integrated in physiotherapy with a duration of 12 weeks. The final intervention is described in more detail below and schematically in Figure 4.

##### Physiotherapy

The physiotherapy treatment was advised to consist of at least eight visits after usual physiotherapy care based on the Dutch physiotherapy guidelines for patients with LBP.^27^

##### Virtual reality

The VR therapy was applied using the Pico Neo 3 (Bytedance, Beijing, China). Patients were instructed to use the VR headset five times a week for 10–30 min at home. Three commercially available VR modules were incorporated in the draft intervention, because of the advantages regarding accessibility and wider applicability. First, Reducept (Reducept, Leeuwarden, The Netherlands; Reducept Game—Play-through demonstration—YouTube) was used for pain education and practicing pain management techniques.

This module contains consecutive sessions of ∼10 min, in which a patient travels through the nervous system and gets exercises based on different psychological treatment principles and pain neuroscience education elements. Second, SyncVR Relax & Distract (SyncVR, Utrecht, The Netherlands; SyncVR Relax & Distract—YouTube) was used for relaxation and distraction. The module contains a wide variety of videos, games, and relaxation exercises with durations varying between 5 and 20 min.

Third, SyncVR Fit (SyncVR; SyncVR Fit—YouTube) was used for general activation. This module includes 11 movement and sports exercises (e.g., tennis and boxing) varying in duration between 5 and 10 min and with three intensity levels. Data security was attended to by not using any personal data on the VR headset and only extracting pseudonymized usability data.

Physiotherapists and patients should select suitable VR modules through shared decision making. Physiotherapists were instructed to start with the VR modules on pain education and relaxation in the 1^st^ week and start using the VR module on activation in the 4^th^ week. Physiotherapists were instructed to discuss the patient's VR use. Based on the patient's experience and preferences with VR, the physiotherapist could adapt (VR) treatment. Also, physiotherapists were advised to prescribe VR exercises for home exercises.

Specifically for those patients with a higher score on pain-related fears (FABQ-PA ≥16 and/or PCS ≥30), physiotherapists were instructed to supplement usual care with exposure therapy. This exposure module was based on four steps: (1) mapping patient-specific pain-related fears, (2) education about these pain-related fears (possibly using the VR module Reducept), (3) facing these pain-related fears (possibly using the VR module SyncVR Fit), (4) facing these pain-related fears in daily life.^50^

## Discussion

### Main findings

This study described the development of a physiotherapy treatment with integrated, multimodal, immersive VR for patients with complex CLBP. Unique for our final intervention is the strong integration of the VR modules in the usual physiotherapy treatment. Previously studied VR interventions were stand-alone interventions with no or minimal guidance of professional therapists.^51–53^ Integration of VR into an existing treatment is challenging and has been reported as an important barrier to adoption.^54^ Therefore, the development of our VR intervention is an important step toward better integrated therapeutic VR.

VR and physiotherapy can strengthen each other as suggested in previous studies^22,23^ and explicitly mentioned in the focus groups in this study. Learned principles through VR education can be made explicit for daily situations in conversation with a trained (physio)therapist, thereby enhancing patients' self-awareness. Vice versa, VR can enhance adherence by making exercising more attractive, improve patient performance, and simultaneously provide possibilities to track patients' performances during exercises.^55,56^

To accomplish this mutually beneficial relationship, VR needs to be administered under the right conditions, and therapists need guidance in the form of a protocol with sufficient freedom to further personalize the intervention through shared decision making. These right conditions were further specified in the focus groups with regard to the technology, physiotherapist, and patient. First, easy-to-use and flawless technology was mentioned in the focus groups, which coincides with results from prior studies on barriers and facilitators to implement VR in physiotherapy.^57^

Second, physiotherapists should have sufficient affinity with and training in therapeutic VR. Prior studies found that therapists administering therapeutic VR could benefit from a multistage training program^58^ and mentioned it as a facilitator for implementing VR in physiotherapy care.^59,60^ In our concept intervention, training of the therapists comprised a single training. Based on the focus groups, this training was extended to two separate 90-min sessions: one specifically regarding VR software and hardware and the other regarding its integration in the physiotherapy treatment and specific research tasks.

This is a one-time and relatively limited time investment and, therefore, regarded feasible and scalable. Third, patients need the right expectations and physical space. This corresponds with previous findings among patients with CMP, which showed that an individual's expectation critically influences the efficacy of pain treatment.^61^

To our knowledge, no previous VR interventions for CLBP were developed using a framework like the MRC framework. This framework provided a systematic approach to develop and test the intervention and the mixed-methods approach allowed us to include a broad range of perspectives from the different stakeholders on the content and design of the intervention while being able to practically test this in a feasibility study. Moreover, the framework includes multiple core elements that were considered during development of the final intervention, such as “context.”

This was taken into account and mentioned during the focus groups as identified in the subtheme that proposed that physiotherapists should have sufficient affinity with and training in VR. This coincides with the recommendation to consider the authentic context of use of digital therapeutics.^62^ Also, intervention refinement is a core element that was included in the development of the final intervention, since multiple adjustments were made to the draft intervention.

To study the added effect of integrated VR in clinical primary care physiotherapy, participants of the focus groups suggested a more pragmatic intervention design. This pragmatic approach may include shared decision making, alternating use of VR modules, and flexibility in the intervention dose. Moreover, the possibility to personalize the intervention was mentioned. This is in line with patient-centered care, as advised for patients with CMP^63^ and shown to improve effectiveness in VR studies.^61,64^ Even though the choice for a more pragmatic approach might limit statements about efficacy and internal validity, the external validity will be higher.^65^

### Strengths and limitations

A main strength of our study is the use of the MRC framework, providing a systematic approach to develop and test the intervention. A combination of homogeneous and heterogeneous focus groups enhanced the participant involvement and idea generation during the group discussions. The end users (patients and physiotherapists) were closely involved in the development and testing of the intervention, which enhances the potential effectiveness and implementation in daily practice. Finally, by using commercially available VR software, implementation in physiotherapy practice is easier and programs are regularly updated.

This study might have benefitted from a larger sample size in the feasibility study. Because of the low sample size, no conclusions can be made about the preliminary effectiveness of the developed intervention. Ideally, a larger pilot cluster RCT would be performed to gain more insight into preliminary effectiveness. Second, the focus of this study was the development of a complex intervention and the examination of its feasibility, rather than comprehensively mapping all possible aspects concerning an integrated VR and physiotherapy intervention.

Instead, we aimed to incorporate the most relevant aspects in the design of the intervention, requiring no data saturation.^66^ Therefore, the iterative study design with five phases and the substantial sample of patients, physiotherapists, and researchers who participated in the focus groups (*n* = 22) was deemed sufficient to give a realistic view on the feasibility of the intervention. Third, we could have explored other topics than the integration of VR and physiotherapy, such as particular elements of the VR therapy in itself. This might have enriched the qualitative data with regard to the benefits and disadvantages of our new intervention.

### Clinical and societal implications

There are many available treatments for CLBP patients. However, the complexity of the medical condition results in generally unsatisfactory outcomes of all of these treatments. Our new intervention, developed together with end users, might be a new tool in the toolbox of health care providers to alleviate the complaints of patients with CLBP. This intervention could potentially support physiotherapists and other health care providers in administering integrated biopsychosocial treatment for patients with CLBP. Moreover, therapeutic VR might improve patients' treatment adherence and enable patients to self-manage their condition.

Although specifically designed for patients with severe disability due to CLBP, it is plausible that this intervention is also applicable to patients with less severe disabilities or other CMP syndromes. However, it should be noted that this intervention is only applicable to patients who are willing and able to use VR. Besides the clinical effectiveness, the VR intervention developed in this study also offers opportunities to reduce health care costs and thereby the worldwide burden of CLBP.^67^ By enabling therapy at home, it may reduce physical visits while enhancing multidisciplinary treatment.

Such home treatment is becoming increasingly important with rising health care costs and additionally reduces the out-of-pocket costs (e.g., travel expenses or informal care arrangements) for patients,^68^ while also offering opportunities for CLBP patients in remote areas.^69^ Fortunately, VR technology is dynamically evolving, with VR systems becoming more affordable and more common, making it increasingly accessible.^70^

## Conclusion

This study described the development of a physiotherapy treatment with integrated, multimodal, personalized, immersive VR for patients with complex CLBP. The intervention development process followed the MRC framework in cocreation with the end users, and resulted in an intervention indicated to be feasible in a small group of patients. Its clinical and (cost-)effectiveness will be further investigated in a separate cluster RCT.

## Abbreviations Used

AEs: adverse events
CLBP: chronic low-back pain
CMP: chronic musculoskeletal pain
FABQ-PA: fear avoidance beliefs questionnaire, physical activity subscale
GPE: global perceived effect
MRC: Medical Research Council
NPRS: pain intensity using the numeric rating scale
ODI: Oswestry Disability Index
PCS: pain catastrophizing scale
PSEQ: pain self-efficacy questionnaire
RCT: randomized controlled trial
VR: virtual reality

## Appendix

**Appendix Table A1.** Moderator Guide Focus Groups

Focus group	Topics
Focus group 1. patients	Intervention design, VR
Focus group 2. physiotherapists	Intervention design, VR, exposure module, integration VR in physiotherapy care
Focus group 3. researchers	Intervention design, VR, exposure module, feasibility study
Focus group 4. combined	Intervention guideline, expectation patients, training physiotherapists, usual care physiotherapy
Focus group 5. patients	General experience, feasibility VR, VR headset and modules, questionnaires, recommendations
Focus group 6. physiotherapists	General experience, training physiotherapists, intake patients, materials, VR headset and modules, integration VR in physiotherapy, contact researcher, recommendations

VR, virtual reality.
